# Supplementary material for: A Rapid and Sensitive Gold Nanoparticle-Based Lateral Flow Immunoassay for Chlorantraniliprole in Agricultural and Environmental Samples
Source: Foods. 2024 Jan 9;13(2):205. doi: 10.3390/foods13020205 (PMC10814772; doi:10.3390/foods13020205)
Supplement: Supplementary file 1 [file foods-13-00205-s001.zip › foods-2723550-supplementary.pdf]

Supplementary Material

A Rapid and sensitive gold nanoparticle-based lateral flow immunoassay for chlorantraniliprole in agricultural and environmental samples

Yanling Wu [a, b], Jiao Li [a, b], Jie Zhu [a, b], Zhaoxian Zhang [c], Shuguang Zhang [d], Minghua Wang [a, b], Xiude Hua [*, a, b]

[a] *College of Plant Protection, Nanjing Agricultural University, Nanjing 210095, China*

[b] *State & Local Joint Engineering Research Center of Green Pesticide Invention and Application, Nanjing 210095, China*

[c] *Key Laboratory of Agri-food Safety of Anhui Province, College of Resources and Environment, Anhui Agricultural University, Hefei 230036, China*

[d] College of Sciences, Nanjing Agricultural University, Nanjing 210095, China

[*] *Corresponding author. E-mail address: huaxiude@njau.edu.cn*

Table S1. IC50 of different anti−CAP mAbs by ic−ELISA

mAb	IC_{50} of mAb (ng/mL)
3G1D10	0.65
2C12D10	0.67
5C5B9	0.24
3H12C8	10.51
1E11E7	1.44

Table S2. Cross−reactivity of analogs structurally related to CAP determined by ic−ELISA

Compound	Structural formula	IC_{50} (ng/mL)	CR (%)
Chlorantraniliprole	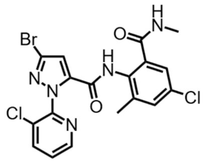	0.24	100
Cyclaniliprole	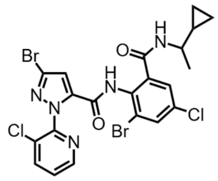	69.9	28.8
Tetraniliprole	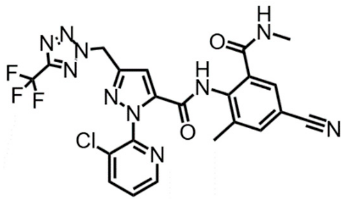	79.8	0.3
Flubendiamide	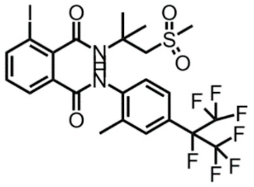	>2000	<0.01
Broflanilide	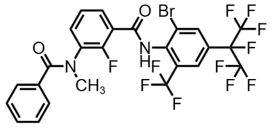	>2000	<0.01
Benzamide	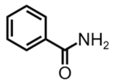	>2000	<0.01
2-(3- chlorine thiophene-2)-3-ketone amino-5-bromine pyrazole	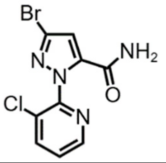	>2000	<0.01

Table S3. The comparison of immunoassays for CAP

Immunoassay	Format	Sensitivity[a] (ng/mL)	Detection time (min)	Reference
ELISA	competitive	1.6	135	[18]
ELISA	competitive	1.5	180	[19]
LFIA	competitive	2.5	15	[32]
AuNP-LFIA	competitive	1.25	10	This work

[a] IC_{50} and visible LOD are used to represent the sensitivity of ELISA and LFIA, respectively.

Table S4. Results of CAP residue detection by UPLC–MS/MS on spiked samples (n = 3).

Spiked (mg/kg)	Recovery (%)					Average recovery (%)	RSD (%)
	1	2	3	4	5		
0.005	97.3	99.8	101.8	103.7	96.9	99.9	2.7
0.02	101.5	99.6	91.4	89.9	91.1	94.6	6.1
0.5	98.9	96.9	100.3	105.7	96.5	99.7	3.7

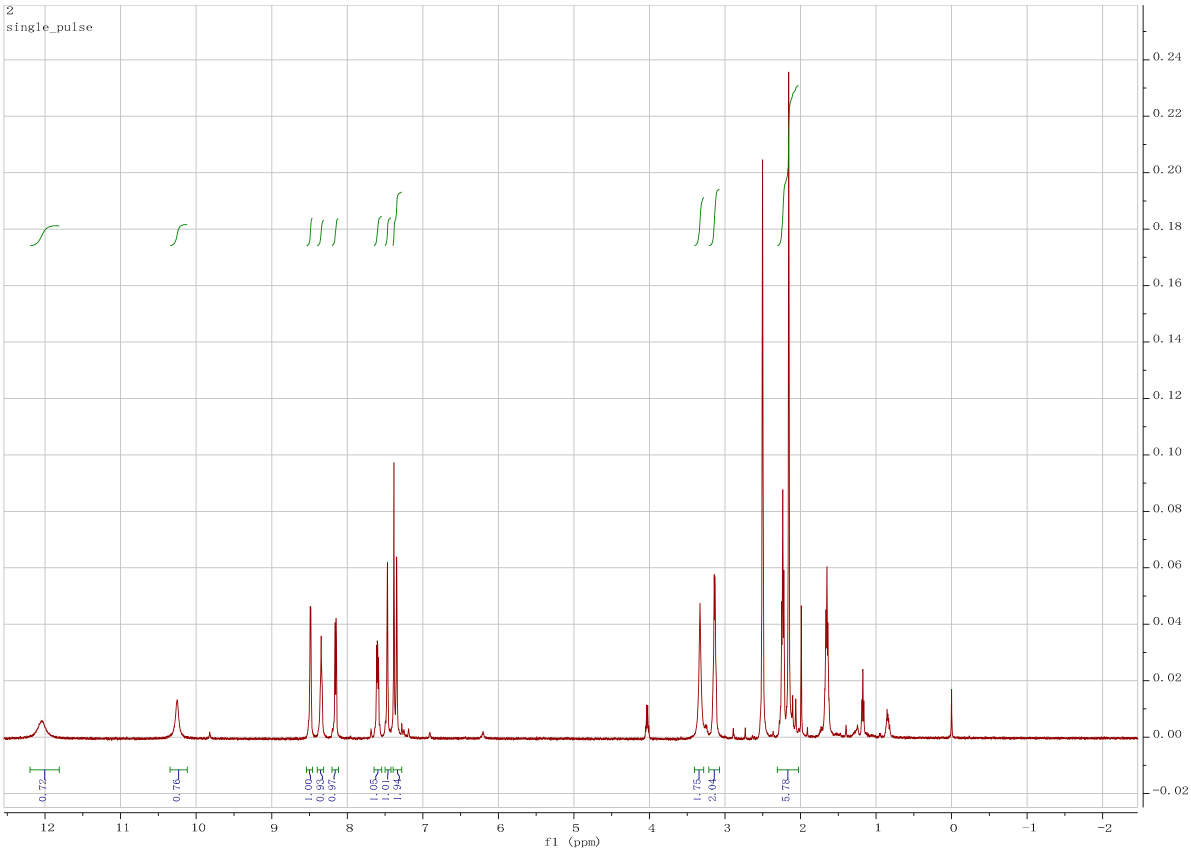

Fig. S1. Illustration of 1H−NMR for CAP hapten

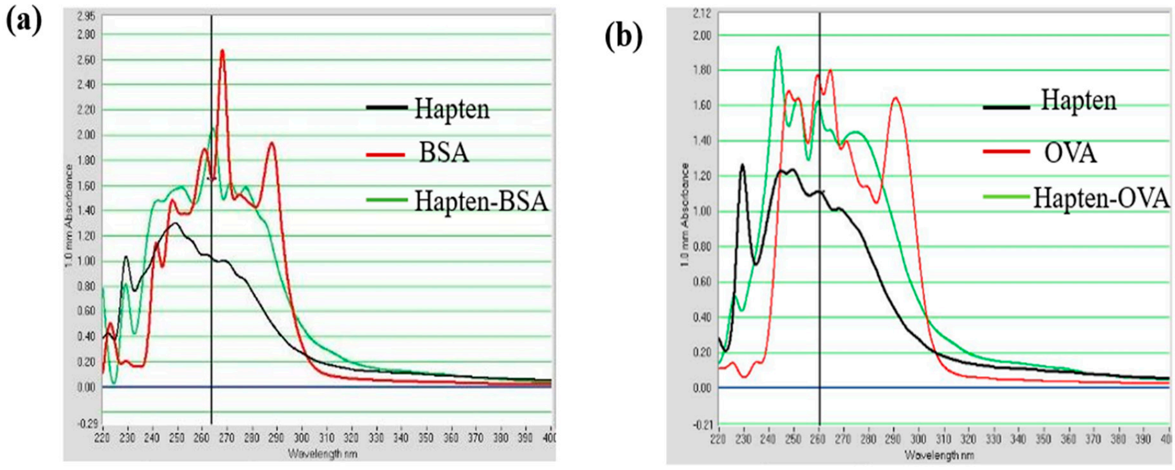

Fig. S2. Ultraviolet absorption spectra of (a) hapten−BSA and (b) hapten−OVA; the concentrations of hap-ten, BSA, OVA, hapten−OVA, and hapten−BSA were 5 mg/mL, 15 mg/mL, 15 mg/mL, 15 mg/mL, and 10 mg/mL, respectively

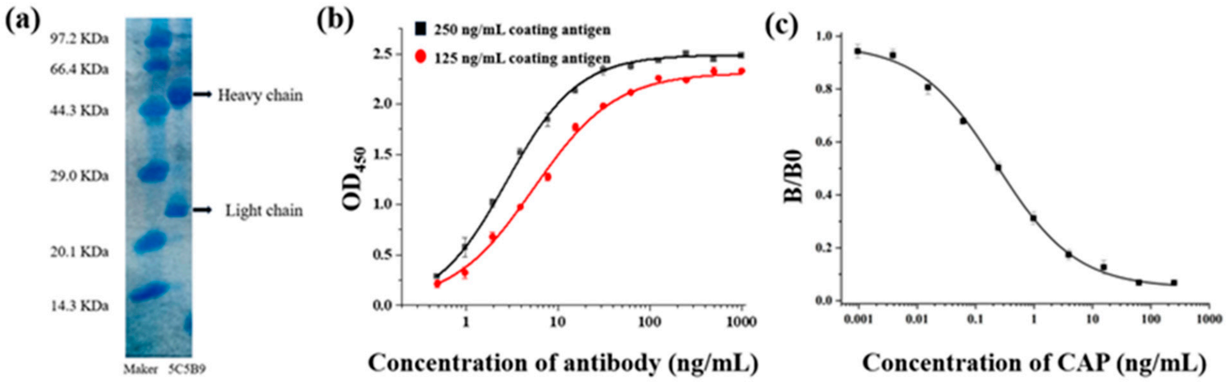

Fig. S3. (a) SDS–PAGE analysis of antibody 5C5B9, (b) Kaff of antibody 5C5B9 by ELISAs, and (c) standard curve of ic–ELISA for CAP

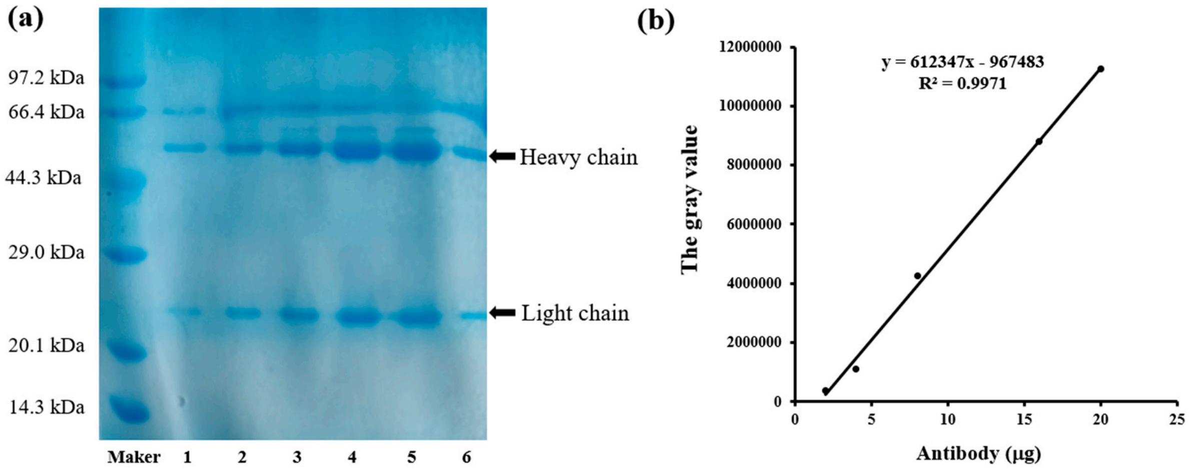

Fig. S4. (a) SDS–PAGE of mAb 5C5B9 and AuNP–labeled mAb; lines 1–5 loaded with 2 μg, 4 μg, 8 μg, 16 μg, and 20 μg mAb 5C5B9, and line 6 loaded with 0.54 mg AuNP–labeled mAb. (b) The standard curve representing the relationship between the gray value of the heavy chain band and the amount of antibody

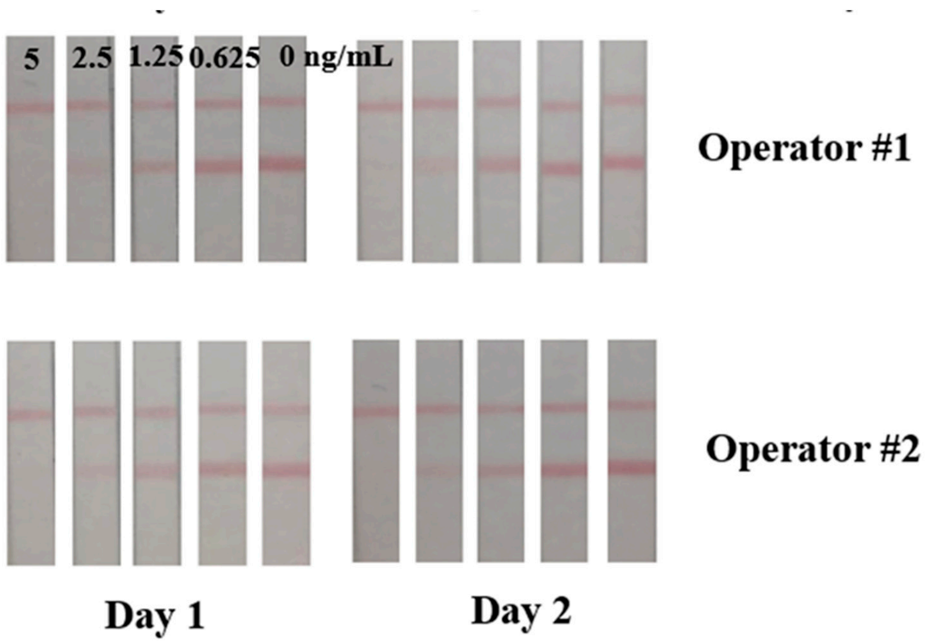

Fig. S5. The sensitivities of the AuNP−LFIA at different detection times with different operators

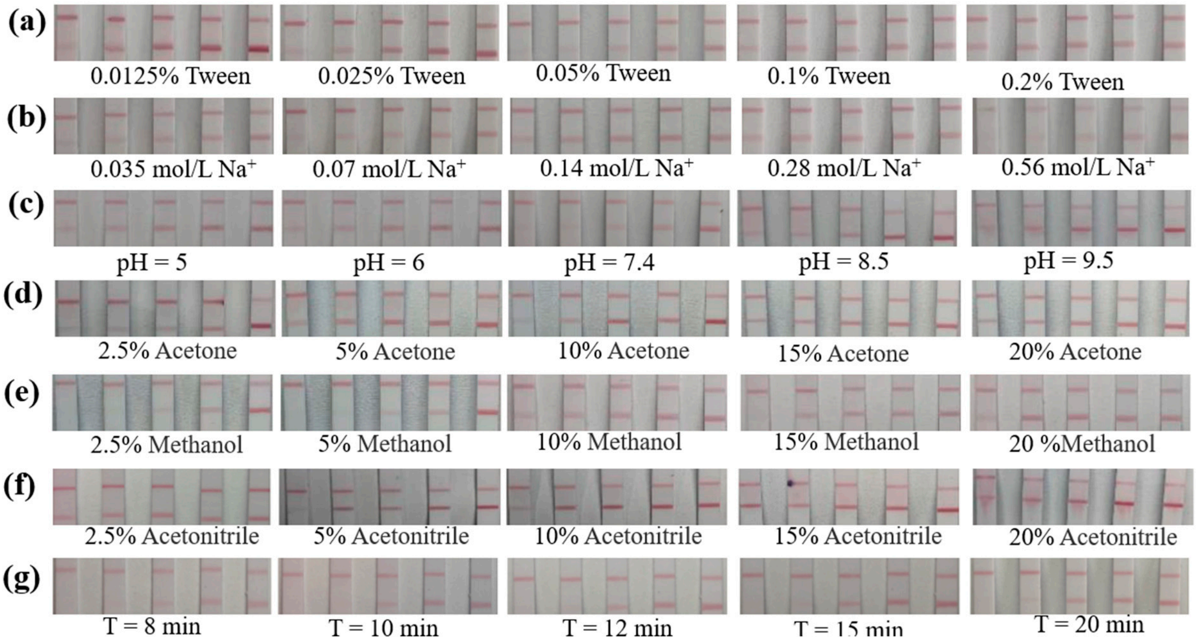

Fig. S6. Optimal parameters for the AuNP−LFIA. (a) Tween−20, (b) Na+, (c) pH, (d) methanol, (e) acetone, (f) acetonitrile, (g) time. CAP concentrations (ng/mL, from left to right): 5, 2.5, 1.25, 0.625, and 0

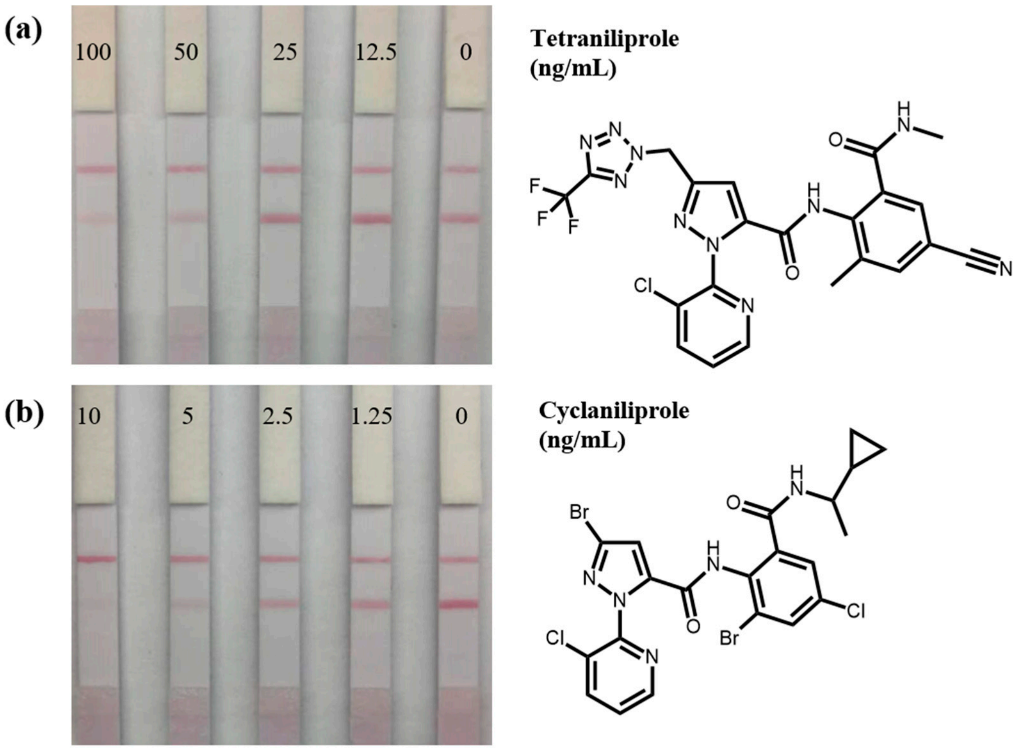

Fig. S7. Images of the AuNP–LFIA for serial concentrations of (a) tetraniliprole and (b) cyclaniliprole

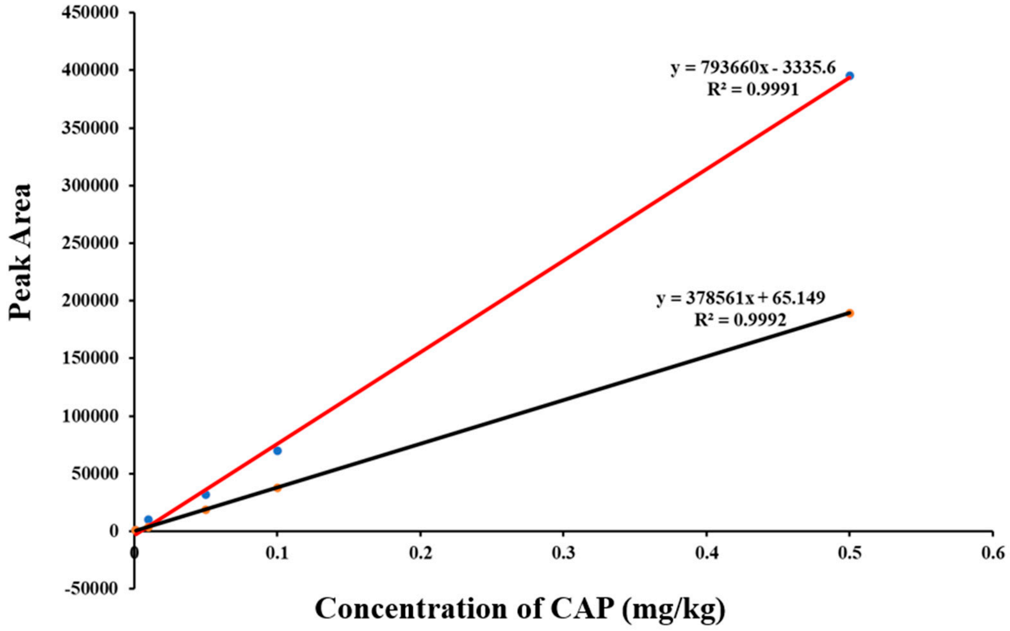

Fig. S8. The solvent standard curve of the solvent and matrix for CAP in brown rice detected by UPLC–MS/MS
